# Microbiota Present in Cystic Fibrosis Lungs as Revealed by Whole Genome Sequencing

**DOI:** 10.1371/journal.pone.0090934

**Published:** 2014-03-05

**Authors:** Philippe M. Hauser, Thomas Bernard, Gilbert Greub, Katia Jaton, Marco Pagni, Gaudenz M. Hafen

**Affiliations:** 1 Institute of Microbiology, Centre Hospitalier Universitaire Vaudois and University of Lausanne, Lausanne, Switzerland; 2 Vital-IT Group, SIB Swiss Institute of Bioinformatics, Lausanne, Switzerland; 3 Department of Paediatrics, Pulmonary Unit, Centre Hospitalier Universitaire Vaudois and University of Lausanne, Lausanne, Switzerland; Charité, Campus Benjamin Franklin, Germany

## Abstract

Determination of the precise composition and variation of microbiota in cystic fibrosis lungs is crucial since chronic inflammation due to microorganisms leads to lung damage and ultimately, death. However, this constitutes a major technical challenge. Culturing of microorganisms does not provide a complete representation of a microbiota, even when using culturomics (high-throughput culture). So far, only PCR-based metagenomics have been investigated. However, these methods are biased towards certain microbial groups, and suffer from uncertain quantification of the different microbial domains. We have explored whole genome sequencing (WGS) using the Illumina high-throughput technology applied directly to DNA extracted from sputa obtained from two cystic fibrosis patients. To detect all microorganism groups, we used four procedures for DNA extraction, each with a different lysis protocol. We avoided biases due to whole DNA amplification thanks to the high efficiency of current Illumina technology. Phylogenomic classification of the reads by three different methods produced similar results. Our results suggest that WGS provides, in a single analysis, a better qualitative and quantitative assessment of microbiota compositions than cultures and PCRs. WGS identified a high quantity of *Haemophilus* spp. (patient 1) or *Staphylococcus* spp. plus *Streptococcus* spp. (patient 2) together with low amounts of anaerobic (*Veillonella*, *Prevotella, Fusobacterium*) and aerobic bacteria (*Gemella, Moraxella, Granulicatella*). WGS suggested that fungal members represented very low proportions of the microbiota, which were detected by cultures and PCRs because of their selectivity. The future increase of reads’ sizes and decrease in cost should ensure the usefulness of WGS for the characterisation of microbiota.

## Introduction

The physiological importance of microbiota present in different localisations of the human body is increasingly recognised and the human microbiota is now considered to be a “human organ” [1]. The composition of microbiota and their variation over time are considered to be involved in the development of infectious diseases (post-antibiotic colitis) and non-infectious diseases (obesity, asthma). The lung microbiota of cystic fibrosis (CF) patients also plays a crucial role in the pathophysiology of the CF disease and this is of major importance since impairment of the lungs is the leading cause of death for these patients. Due to the pathophysiological defect of the CF transmembrane conductance regulator at the apical surface of the airways, CF lung disease is characterised by a vicious cycle of chronic airways inflammation and infection that leads to the injury of the lung parenchyma with progression to premature death. Healthy lungs were considered to be sterile until recently. There is now increasing evidence that the bronchi harbour a specific microbiome in healthy individuals as well as in CF patients [2], [3]. The currently used antibiotic treatments have incontestably increased the life expectancy of CF patients. Nevertheless, these combinations of antibiotics may also cause “collateral” damage due to the development of resistance, with the consequence of no further significant increase of life expectancy. More therapeutic options for CF lung disease could become available in the future, by targeting other pathogens than *Staphylococcus aureus*, *Pseudomonas aeruginosa*, and *Aspergillus fumigatus*, by discovering new therapeutic strategies, like for example adapting lung environments [4], or by identifying beneficial probiotic microorganisms. For that purpose, it is important to have a holistic view of the lung microbiota of CF patients, including not only bacteria but also fungi and viruses. This holistic view is also needed because there is evidence of signalling interactions between species of a given biological domain (bacteria or eukarya), but also between species of different domains, either as pathogens, commensals or synergetic microorganisms [5].

Determining the composition of CF lung microbiota is not straightforward and constitutes a major technical challenge [6], [7]. Indeed, the media used in classical microbial culture methods only allow identification of cultivable microorganisms and of selected groups of microorganisms. Even using culturomics, there is still a huge gap between recovered microbes and those detected by metagenomics [8], [9]. Obviously, cultivable microorganisms only correspond to a small fraction of the total microbiota. Therefore, recent studies investigate the usefulness of molecular biology to obtain a more comprehensive composition of microbiota [10].

PCR amplification of the genes encoding the ribosomal RNAs followed by high-throughput DNA sequencing (HTS) has been largely used so far because large databases of these sequences exist. These studies almost exclusively targeted bacteria using 16S rRNA [2], [11], [12], [13], [14], [15] and only few studies analysed fungi using the internal transcribed spacers (ITS) [16], [17]. The data presently available suggest a bacterial diversity of the CF lung microbiota that increases in childhood [11], followed by a progressive decline during adulthood [12]. A greater diversity appeared to be associated with better lung functions. The most common organisms observed in CF lungs are *S. aureus, Haemophilus influenzae, P. aeruginosa*, *Burkholderia cepacia,* and *A. fumigatus*. The clinical importance of *A. fumigatus* and other fungi in CF remains controversial. Nevertheless, fungi and bacteria are known to interact at the cellular level [18], specifically *P. aeruginosa* with *A. fumigatus* [19], or *P. aeruginosa* with *Candida albicans* [18].

However, the PCR approach used so far suffers from several limitations and may introduce biases in the observed microbiota compositions. Indeed, selected groups of organisms will be preferentially amplified by the primers, as demonstrated for the fungal ITSs [20]. Even broad-range eubacterial 16S rRNA PCR generally do not amplify all eubacteria, since Spirochetes and Chlamydia for instance exhibit highly divergent sequences. PCR also implies the risk of missing as yet unknown microorganisms which are not amplifiable because of alternate target sequences. Consequently, running several PCRs in parallel is necessary to obtain a comprehensive composition of the microbiota, but this prevents a relative quantification of the different microorganism groups between each other. There is thus a need to improve the determination of the composition of microbiota in order to get quantitative and complete results. The aim of the present work was to assess the usefulness of whole genome sequencing (WGS) using the Illumina HTS technology applied directly to DNA extracted from CF respiratory clinical samples.

## Materials and Methods

### Ethics Statement

We obtained informed consent from the two 14 and 17 year old female patients as well as from their next of kin. The obtained consent was verbal in agreement with the institutional review board, the Ethics Committee of the Canton of Vaud, Lausanne, Switzerland. Written consent was not necessary because the analysis was performed using left-overs of clinical samples. The oral consent was recorded in the patients’ medical documentation. The institutional review board approved the study protocol (No. 352/11).

### Specimens

Two spontaneous sputa were collected in 2012 from adolescent female CF patients at the paediatric outpatient clinic of the Centre Hospitalier Universitaire Vaudois. They were processed on the day of collection for cultures in the microbiology diagnostic laboratory, and then stored at −20°C before DNA extraction.

### Cultures

The routine procedure for culturing respiratory specimens from CF patients was applied in the microbiology laboratory. Briefly, five different agar media were used in Petri dishes. All media are from Becton Dickinson (Allschwil, Switzerland), except the MacConkey medium which is from Biolife (Milano, Italia). The MacConkey medium was incubated for 48 hours in an aerobic atmosphere, whereas the Columbia and Chocolate-bacitracin agar were incubated in a 5% CO_2_ atmosphere. Specific cultures were carried out to detect *B. cepacia* at 37°C for 72 hours in an aerobic atmosphere on the Oxidation/Fermentation-Polymyxin-Bacitracin-Lactose (OFPBL) agar. Bacterial growth was scored semi-quantitatively for each species, and each strain was identified using conventional procedures. Species considered as possible pathogens (e.g. *H. influenzae, S. aureus, Achromobacter* sp. and *P. aeruginosa*) were identified using matrix-assisted laser desorption ionisation time-of-flight (MALDI-TOF) mass spectrometry using a cut-off of 2 for identification at species level, and of 1.7 at genus level [21]. MALDI-TOF proved to be a very good tool for identifying non-fermentable bacteria. Filamentous fungi and yeasts were cultured at 30°C for up to seven days in an aerobic atmosphere on the Sabouraud medium supplemented with 0.5 g/l chloramphenicol and 0.04 g/l gentamicin to prevent bacteria growth. Growth of yeasts and *Candida* spp. were scored semi-quantitatively, using the four-quadrant method. Filamentous fungi growth was scored by reporting the total number of fungal colonies, and the colonies were identified morphologically upon sporulation on potato dextrose medium (Becton Dickinson).

### DNA Extraction using Four Methods in Parallel

To avoid contamination, DNA extraction was performed under a laminar flow situated in a different room from where subsequent PCR amplification and sequencing steps were carried out. Each sputum was liquefied by the addition of one volume of fresh 2% w/v N-acetyl-L-cystein in Tris-Na-citrate-di-hydride 1.5% w/v pH 8.5, and by vortexing and agitating for at least 30 minutes on a rotating agitator. After centrifugation at 3000 g for 15 minutes, the pellet was resuspended in a volume of supernatant equal to the initial volume of the sputum. To improve the recovery of genomic DNA and thus to permit an exhaustive evaluation of the microbial diversity, four methods of lysis were used. Practically, the liquefied sputum was separated in four aliquots of 200 microlitres: one was used for direct DNA extraction (see below), and three for the following different lysis procedures performed before DNA extraction. One aliquot was incubated for 15 minutes at 95°C followed by incubation in a sonication bath for 10 minutes (procedure for mycobacteria of our molecular diagnostic laboratory). The two other aliquots were subjected to beads-beating for 30 or 45 seconds: after the addition of an equal volume of Bacteria Lysis Buffer and brief vortexing, each aliquot was transferred to a dedicated tube containing MagNA Lyser green beads, and shaken at 6,000 rpm in the MagNA Lyser agitator (reagents and device: Roche, Basel, Switzerland; procedure for fungi of our molecular diagnostic laboratory). The tubes were then centrifuged for 10 minutes at 10,000 rpm, and 100 microlitres of the supernatant were transferred into a new tube. DNA was then extracted using a QIAamp DNA mini-kit (including lysis with proteinase K, Qiagen: Hilden, Germany) according to the manufacturer’s instructions. The four DNA extracts were mixed in equal amounts. The resulting DNA mixture from sputa 1 and 2 were 66 and 312 ng/ml respectively.

### WGS by Illumina HTS

Single-end libraries were prepared using Illumina TruSeqDNA Sample Prep reagents and 1.3 or 1.4 micrograms of the DNA mixture obtained from sputa 1 or 2 respectively. Multiplex sequencing produced 95,507,767 and 101,556,686 reads respectively with an average length of 100 nucleotides.

### Illumina HTS Read Filtering

Prior to any analysis, most of the human reads were filtered out of the two datasets by aligning all the reads onto the UCSC hg19 human genome using Bowtie [22] with a seed of 75 and allowing one mismatch. This reduced the number of reads to 16,233,617 (17.0%) and 20,191,018 (19.8%) for sputa 1 and 2 respectively. The metagenomics data were not deposited in a publicly available database because they contain information (human genome reads) that could allow identification of the patients, even after filtration. Nevertheless, the data are available for research purposes upon request.

### Search for Bacterial Sequences among Filtered HTS Reads

Phylogeny analyses of the unassembled Illumina reads were then performed using BLAST against SILVA rRNA library [23], as well as using MetaPhlan [24] and MetaPhyler [25], according to the instructions. We considered only reads that could be attributed to a bacterial taxon and representing at least 0.1% of the whole population of microbial genera/species observed in a given microbiota.

### Search for Fungal Sequences among Filtered HTS Reads

We searched for fungal ITSs of the rRNA operon in the non-filtered datasets using BLAST against the ITSoneDB [26] and UNITE [27] databases for ITS1, and against the ITS2 database IV [28] and UNITE databases for ITS2. We also searched for homology between the reads and all currently available fungal proteins in UniProt using blastx [29]. As these protein searches were computationally demanding on the complete datasets, we only performed them on 1% of the filtered datasets. As an additional control, we used Bowtie (seed of 75, 1 mismatch) to map the reads of the two non-filtered datasets on different genomes of the genus *Candida* (www.candidagenome.org), and of the genus *Aspergillus* (http://www.aspergillusgenome.org).

### Eubacterial PCR Targeting 16S rRNA

The eubacterial PCR described by Delhaes et al [16] was used. It amplifies a ca. 465 bps region containing the complete V3 domain of most prokaryotic 16S ribosomal DNA genes. This PCR is known to amplify more than 95% of the species of all eubacterial phyla, except for the following phyla of which only 5 to 90% of the species are amplified: Chlamydiae, Chlorobacteria, Cyanobacteria, Planctomycetes and Verrucomicrobia [30]. The primers were 3271-16S-F (TACGGRAGGCAGCAG) and 3271-16S-R (GGACTACCAGGGTATCTAAT). PCR was performed with two microlitres of sputum DNA mixture in a final volume of 20 microlitres containing 0.35 U High-Fidelity Expand Polymerase (Roche), the provided Expand buffer, each dNTP at 200 microM, each primer at 4 microM, and a final MgCl2 concentration of 3 mM. An initial denaturation step of 3 minutes at 94°C was followed by 40 cycles consisting of 30 seconds at 94°C, 30 seconds at 50°C, and 30 seconds at 72°C. The reaction was terminated with 10 minutes of extension at 72°C. All PCRs were prepared under a laminar flow hood located in a different room from where the PCRs were analysed. PCRs were carried out using a Techne TC-312 thermocycler, model FTC3102D. The PCR products were analysed in a 1% agarose gel containing Ethidium bromide (0.5 microg/ml).

### Pan-fungal PCR Targeting ITS2

The pan-fungal PCR described by Delhaes et al [16] was used. It amplifies a 340 to 360 bps fragment of the ITS2 region from all major fungi phyla. The primers were 3271-ITS2F (CARCAAYGGATCTCTTGG) and 3271-ITS2R (GATATGCTTAAGTTCAGCGGGT). The PCR conditions were exactly the same as used for the 16S rRNA eubacterial PCR (see above). Pan-fungal PCR products were only obtained after random amplification of the sputa DNA mixtures. Random amplification was carried out using two microlitres of sputum DNA mixture and the Illustra GenomiPhi V2 DNA Amplification Kit according to the manufacturer’s instructions (GE Healthcare, Glattbrugg, Switzerland). As a control, *Saccharomyces cerevisiae* genomic DNA was readily amplified without previous random amplification, suggesting that the ITS2 primers performed properly.

### Real-time PCR for *A. fumigatus*

The real-time PCR specific for *A. fumigatus* amplified a 85 bps fragment of the mitochondrial cytochrome B. The pipeting TECAN EVO 150 robot (8 channels) was used to prepare a reaction mixture containing master mix ABI Taqman universal PCR, primer AFF (TTGTATTCTTCATGCCTAACGCA), primer AFR (CGGAACAATAGCAGGTGGAGTT), and the probe (FAM-AGGTGATAGTGAAAATTATGTTATGGCTAATCCAATGC-BHQ1). Each PCR reaction included 15 µl of reaction mixture and 5 µl of sputum DNA mixture. Real-time PCR was performed using Taqman 7900 (Applied Biosystems, Zug, Switzerland). After 2 minutes at 50°C and 10 minutes at 95°C, 45 cycles of 15 seconds at 95°C and 1 minute at 60°C were performed. The conversion of Cts in the target concentration was obtained using a calibration curve obtained by amplifying successive dilutions of a plasmid containing the *A. fumigatus* target sequence. The absence of inhibition was tested by a reaction containing the tested DNA and 1,000 copies of the plasmid containing the target. Each sample was analysed in duplicate. The limit of detection was one to ten copies of the target per reaction.

### Cloning, Sequencing and Classification of PCR Sequences

The PCR products were extracted from the gel using a QIAquick Gel Extraction Kit (Qiagen, Hilden, Germany), and sub-cloned using a TOPO TA cloning kit and One Shot DH5alpha-T1 competent cells according to the manufacturer’s instructions (Invitrogen, Life Technologies, Lucern, Switzerland). They were sequenced using a reverse M13(-20) primer, Big Dye Terminator DNA sequencing kit, and an ABI PRISM 3100 automated sequencer (both from Perkin-Elmer Biosystems, Rotkreuz, Switzerland). Eubacterial 16S rRNA sequences were identified by comparison using BLAST against two databases of the National Centre for Biotechnology Information (NCBI, www.ncbi.nlm.nih.gov): “reference RNA sequences” and “16S ribosomal RNA sequences (bacteria and archea)”. Both databases provided identical results at the genus level. For pan-fungal ITS2 sequences, the NCBI “nucleotide collection” database was used. Sequences were classified to a genus-level using a 95% global identity threshold. The procedure of PCR followed by sub-cloning and sequencing was repeated twice. The sequences of PCR clones were not deposited in a public database because of their limited quality resulting from single strand sequencing.

## Results

### Rationale

The Illumina HTS technology was chosen for WGS because (i) it requires small amounts of DNA, avoiding random DNA amplification, a technique which may introduce biases in the proportions of the different microbial species, (ii) it produces large amounts of reads which ensures analysis of the microbiota despite the presence of a large proportion of human DNA (about 80%), and (iii) it is less expensive than other technologies, allowing analysis of more samples as required for future studies. We used several different DNA extraction procedures to decrease possible bias related to the different cell wall structures, and we pooled the DNA extracts before direct Illumina HTS and PCRs. This was meant to ensure the detection of as many different groups of microorganisms as feasible, and thus improve the enumeration of microbial diversity. Routine microbiological cultures, as well as eubacterial and pan-fungal PCRs, followed by sequencing sub-clones, were applied to the same DNA preparations for comparison.

### Dataset Processing

To reduce the size of the DNA sequence datasets obtained from the two CF sputa, most human reads were filtered out. The filtering parameters were designed to ensure that all non-human reads were kept. The accuracy of the phylogenomic classification methods increasing with the size of the reads, we attempted to assemble our filtered reads into contigs. Both tested programs (Velvet [31] and SPAdes [32]) assembled only ca. 15% of the reads into small contigs (70% <1 kb). This poor assembly performance is clearly linked to the short length of the reads investigated, as well as to the great mixture of organisms present in our samples (see below). Considering the minor improvement in sequence length and added risk of creating chimeric contigs, we decided to perform all further analyses using the unassembled raw reads.

### Phylogenomic Classification

To determine the composition of the microbiota, we used three different methods of phylogenomic classification. First, ribosomal DNA sequences were searched using BLAST against the SILVA rRNA library, and a lowest common-ancestor approach was used to assign a taxon to each matching read. The two other methods, MetaPhlan and MetaPhyler, consisted in searching for clade-specific marker genes. In addition, we tried the composition-based method TAXY designed to be used with short reads [33], but it did not produce convincing results. Indeed, different results were obtained whether it was applied on complete or filtered datasets, which was not the case with the other three methods.

### Populations of the Observed Microbial Genera

**Figure 1:**
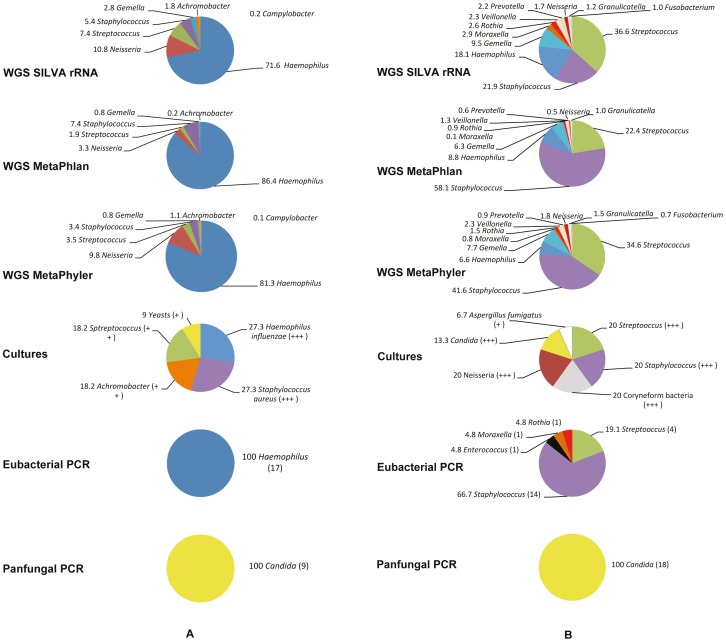
Populations of microbial genera/species observed in CF sputa using different methods. The percentage of the whole population is given for each genus/species. **WGS:** DNA extracted from sputa was sequenced using Illumina high throughput technology and the reads were attributed to a bacterial taxon using three phylogenomic classification methods. **Cultures:** routine procedure for culturing respiratory specimens from CF patients was applied. Low (+) to high (+++) quantity is given in parentheses for each genus/species. *Streptococcus* spp., *Neisseria* spp., Coryneform bacteria, *C. albicans,* and yeasts were considered as part of the oropharyngeal flora; thus, not all were identified at the species level. **PCRs:** PCR products obtained from DNA extracted from sputa were subcloned and sequenced. The number of clones sequenced is given in parentheses for each genus; it is the total of two separate experiments. (**A**) Sputum 1. (**B**) Sputum 2.

The populations of microbial genera found in the two CF sputa using WGS, cultures, and PCRs are represented in Figure 1. The short Illumina reads and the sequences from the PCRs allowed for confident identification only at the genus level. For the two samples, the three methods of read classifications provided similar bacterial microbiota compositions.

### Bacterial Members of the Microbiota

As far as bacterial members of the microbiota are concerned, the results of WGS were consistent with those of the cultures and eubacterial PCR for dominant genera: sputum 1 was enriched in *Haemophilus* spp., whereas *Staphylococcus* spp. and *Streptococcus* spp. predominated in sputum 2. The abundant “Coryneform bacteria” observed in the cultures from sputum 2 include the genus *Rothia* and thus may correspond to the 0.9 to 4.8% of WGS and PCR sequences attributed to this genus. However, some genera evidenced by WGS were not detected by cultures and PCRs, *i.e. Neisseria* spp. in sputum 1, and *Haemophilus* spp. plus *Gemella* spp. in sputum 2. In contrast, WGS detected all genera evidenced by cultures and PCRs, although sometimes in lower proportion (*e.g. Staphylococcus* spp. in sputum 1), or in higher proportion (*Haemophilus* spp. in sputum 1). WGS also evidenced in both sputa minorities of anaerobic genera (*Veillonella*, *Prevotella, Fusobacterium*), as well as other genera (*Gemella, Moraxella, Granulicatella*) which were not specifically searched by cultures, and which were not detected by PCR, possibly because we analysed relatively few sub-clones. One eubacterial PCR sub-clone from sputum 2 corresponded to an *Enterococcus* sp., and might not have been differentiated from *Streptococcus* spp. by WGS because of the short reads.

### Fungal Members of the Microbiota

As far as the fungal members of the microbiota are concerned, cultures detected yeasts in sputum 1, and *C. albicans* plus *A. fumigatus* (14 colonies) in sputum 2 (Figure 1). Pan-fungal PCRs detected only *Candida* in both sputa, suggesting that the yeasts evidenced in the cultures from sputum 1 belonged to that genus. Notably, in contrast to the eubacterial PCRs, pan-fungal PCRs necessitated previous random amplification of sputa DNA to obtain products, suggesting a low fungal load. Nevertheless, both sputa were positive using a real-time PCR specific for *A. fumigatus* cytochrome B, evidencing approximately 34 and 9,500 cells per ml of sputum for sputa 1 and 2 respectively (considering ten mitochondria per haploid cell). On the other hand, WGS did not permit the detection of any fungi. Because of this apparent discrepancy, an extensive search for fungal sequences in the non-filtered Illumina read datasets was performed. First, we searched for fungal ITSs by BLAST against the relevant databases. Second, we looked for homology between the reads and all currently available fungal proteins. The two methods did not provide any significant clues to the presence of fungal sequences. In a complementary attempt, the non-filtered datasets were mapped on different genomes of the genus *Candida* and on the human genome. Only 203 and 1,533 reads for sputa 1 and 2 respectively aligned to a *Candida* genome but not to that of *Homo*. The same analysis using different genomes of the genus *Aspergillus* respectively identified only 230 and 195 reads. We concluded that the *Candida* or *Aspergillus* sequences were possibly present but represented only an extremely low proportion of all reads present in the two datasets (<0.001%).

## Discussion

To determine the composition of microbiota present in CF lungs using WGS, we used three methods of phylogenomic classification which gave similar results, biases due to random DNA amplification were avoided, and several DNA extraction procedures were used, providing confidence in the microbiota compositions obtained. As far as bacteria are concerned, our results suggest that these compositions are much more comprehensive than those obtained by routine cultures and eubacterial PCR. Indeed, several genera which are easy to grow and were abundant according to WGS were not evidenced by these former methods. Moreover, several genera detected by WGS, although in low amounts, would have required specific culture conditions or deep sequencing of the PCR products to be detected. As far as fungi are concerned, WGS suggested that they were at extremely low concentrations in the two microbiota, whereas cultures and PCRs detected them. This discrepancy is probably not due to the non-extraction of fungal DNA because positive pan-fungal PCRs and *A. fumigatus* qPCR were obtained from the same DNA preparations. In addition, the beads-beating DNA extraction procedure used has been specifically developed for fungi and its efficiency has been observed during the last two years on about 200 samples for diagnosis purpose. On the other hand, culture and PCR approaches can detect tiny numbers of fungal cells representing very small proportions of the population because both are very sensitive as they were developed specifically to detect fungi, *i.e.* the culture relies on a selective medium inhibiting bacterial growth, and the PCR primers were designed to amplify only fungal sequences. A low fungal load in the two sputa is also suggested by the fact that DNA had to be randomly amplified to obtain PCR products with pan-fungal PCR.

The composition of the two cystic fibrosis microbiota obtained using WGS is consistent with those previously reported in paediatric CF patients, *i.e.* with a predominance of *H. Influenzae* [10], of *Staphylococcus* [34], or of *Streptococcus* [11]. Most bacterial genera revealed by WGS to be present in low amount in our work have been previously detected using PCR in CF lungs (*Veillonella, Prevotella, Gemella, Moraxella, Rothia, Fusobacterium*) [11], [13], [14], [16]. Previous studies using PCR suggested that *Gemella* might play a direct role in and/or be a biomarker for the exacerbation of lung inflammation in CF patients [13]. The *Streptococcus milleri* group spp. have also been reported to be associated with such episodes of exacerbation [35]. However, these observations remained controversial because the latter association was not confirmed [13], and another study suggested that the same *S. milleri* group spp. play an important role in increasing the biodiversity of CF lung microbiota, promoting patient stability [14]. A varying abundance of these bacteria has been suggested to play a role in this controversy [14]. These conflicting observations further underscore the crucial need for a robust and holistic method providing a qualitative and quantitative characterisation of microbiota in CF lungs.

Our study presents several limitations. First, despite using several DNA extraction methods in parallel to detect as many microbial groups as possible, we cannot exclude biases in the proportions of microbiota members due to varying efficiencies of DNA extraction. This issue will require the analysis of samples spiked with a precise amount of a given species and/or the analysis of large numbers of samples known to contain a given amount of bacteria and fungi, as determined by another reference method, such as CFU determination. Such experiments will help to assess extraction efficiency for different microorganisms, and to improve the procedure. Second, uneven sequencing coverage resulting from varying GC content may have led to incorrect estimation of the abundance of some members of the microbiota, although the Illumina technology has been reported to be one of the less biased in this respect [36]. Third, the shortness of the reads may have provoked some imprecise classifications which may explain differences in the proportions of some genera between the three methods of classifications, *e.g. Neisseria* spp. and *Haemophilus* spp. in sputa 1 and 2, respectively. Fourth, our WGS procedure also sequenced DNA from dead cells, which may mask variations in microbiota composition. This can be avoided by the use of propidium monoazide (PMA), which only destroys the DNA of dead cells because this toxic compound is excluded from viable ones [15]. Comparison with and without PMA treatment might prove useful in the future to understand CF microbiota evolution. Another limitation is that we did not look for reads from DNA viruses. Viruses may play an important role in CF lung disease [37], and we plan to analyse them in the future. Finally, the use of spontaneous sputa rather than bronchoalveolar lavage (BAL) might constitute a limitation because of a possible contamination of sputa by upper-airway flora. There is an ongoing debate with conflicting results about the sample to use in order to obtain the best representation of lung microbiota: spontaneous sputum, induced sputum, throat swabs, and/or BAL. BAL presents the major problem in that it generally cannot sample all the different microbial communities which are compartmentalised within different areas of CF lungs [38]. Moreover, BAL is invasive and has a technical requirement, particularly for repeated assessment. In children there is, in addition, the need for a general anaesthesia. In the view of the latter issues and to prepare our future studies in paediatric patients, we chose to exploit sputa. To improve the quality of sputa, we intend to reduce the oral flora using mouthwash with sterile saline 0.9% prior to expectoration.

Our results suggest that WGS using direct Illumina HTS provides, in a single analysis, microbiota compositions which are more comprehensive and better cover the different taxonomic diversity than any other approaches. Moreover, the ever-increasing read lengths should allow identification of these microbes at the species level in the near future. Importantly, the approach endows few biases, as well as less risk of contamination because of fewer manipulations than PCR-based approaches. The cost is presently high, but it is expected to decrease, and can be further reduced by the opportunity to multiplex. Thus, together with an improvement in phylogeny classification pipelines, it should soon be feasible to analyse many samples quickly and at a low price. In conclusion, it is likely that WGS using direct HTS will prove useful for studying the evolution of CF lung microbiota over time, and this may prove very important to improve care for CF patients suffering from exacerbations. Moreover, such a direct WGS approach will also prove useful in studying other microbiota.
